# Nerve-suturing

**Published:** 1896-07

**Authors:** W. E. A. Wyman

**Affiliations:** Clemson Agricultural College and South Carolina Experiment Station


### NERVE-SUTURING.

By W. E. A. WYMAN, V.S.,
CLEMSON AGRICULTURAL COLLEGE AND SOUTH CAROLINA EXPERIMENT STATION.

Some time ago the writer was called to see a filly which, in a runaway, had struck the side of her head against an oak post, and was bleeding to death. On arrival, a wound about six inches in length, extending from the inferior border of the temporal muscle to the middle of the masseter, a copious hemorrhage from that wound, and the upper lip drawn over to the left side were noticed. As the animal was of a very excitable disposition, she was thrown down, the examination revealing the following facts: The zygomatic arch was crushed to an extent of about one inch, this fracture being about in the middle of the arch. The transverse facial artery and vein were severed and the facial nerve completely cut in two, and some fibres of the uppermost layer of the masseter torn across. All this in line with the fractured portion of the zygomatic arch. After ligating the bloodvessels the writer irrigated the wound for about five minutes with a 1:8000 bichloride of mercury solution; next removed all the splinters of bone and also a little of each side of the compound fracture of the zygomatic arch, as the edges after removal of the bony splinters appeared distinctly serrated. The torn fibres of the masseter were snipped off and the wound irrigated once more with the mercury solution. The facial nerve was sutured with very fine catgut, an effort being made to get the stitches into the connective tissue holding the little bundle of nerve-fibres together. The whole wound was irrigated once more, all moisture removed with "tupfers" as far as possible, and the whole wound thickly dusted with acetanilid-iodoform-tannin, 1 : 1 : 3.

Of course, all the instruments as well as everything coming in contact with the wound had been sterilized previously, and the irrigations used in order to change the infected wound if possible into an aseptic one. The animal was kept tied up for one week; at the fifth day the upper lip had regained its tonicity to such an extent that only the experienced eye could detect a slight deviation toward the left. The filly made an uneventful recovery, being at work eighteen days after the runaway.

